# Supplementary figures and images for: NEAL: an open-source tool for audio annotation
Source: PeerJ. 2023 Aug 25;11:e15913. doi: 10.7717/peerj.15913 (PMC10461540; doi:10.7717/peerj.15913)

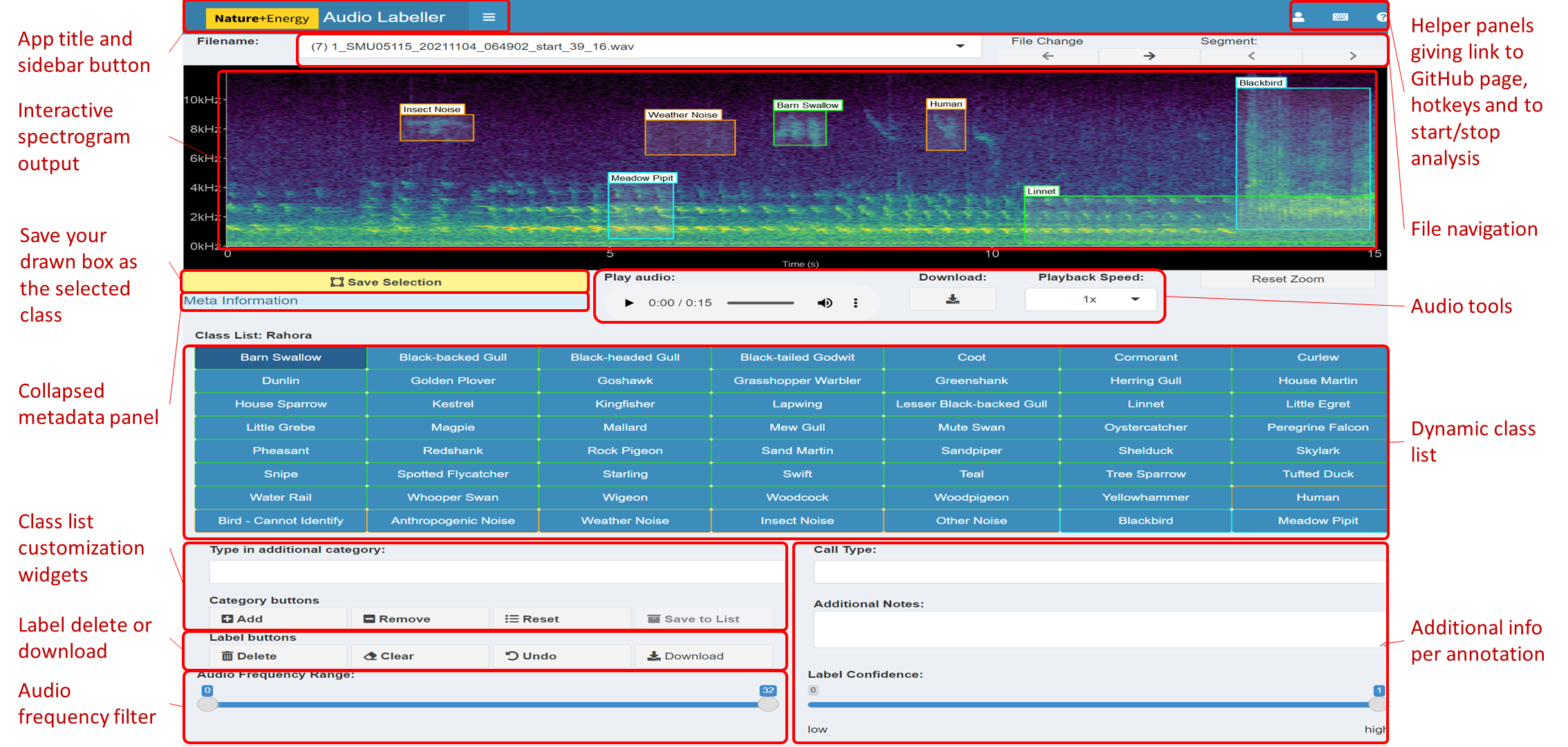

Supplement: Supplemental Information 1 [file peerj-11-15913-s001.png]

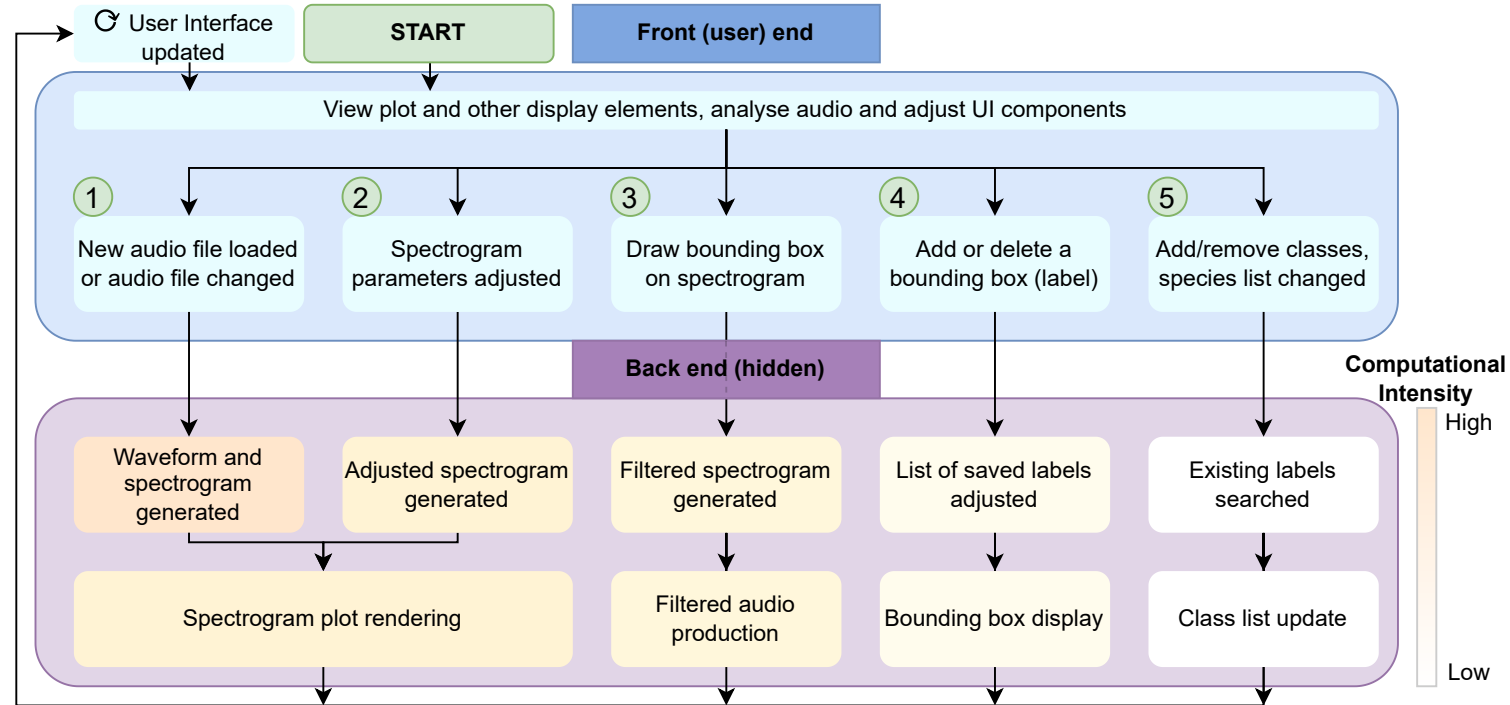

Supplement: Supplemental Information 2 [file peerj-11-15913-s002.pdf]

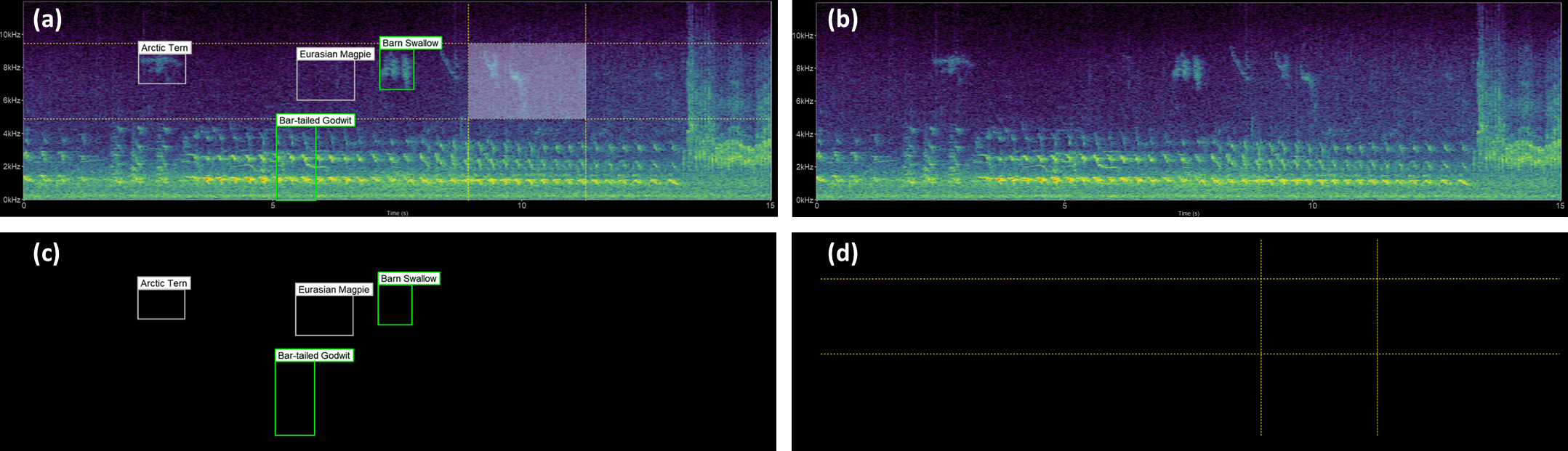

Supplement: Supplemental Information 3 [file peerj-11-15913-s003.png]

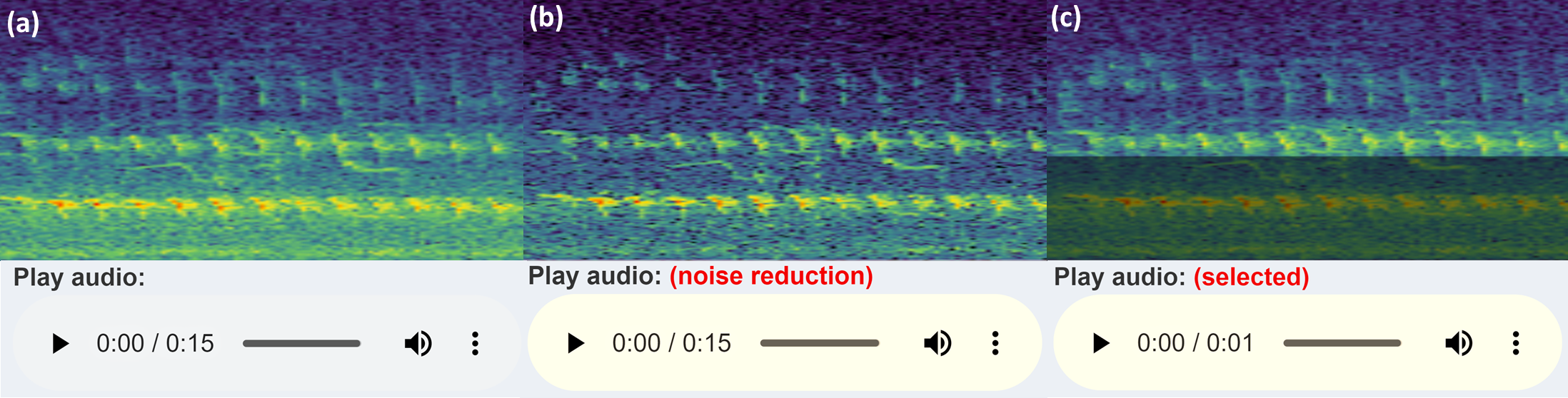

Supplement: Supplemental Information 4 [file peerj-11-15913-s004.png]

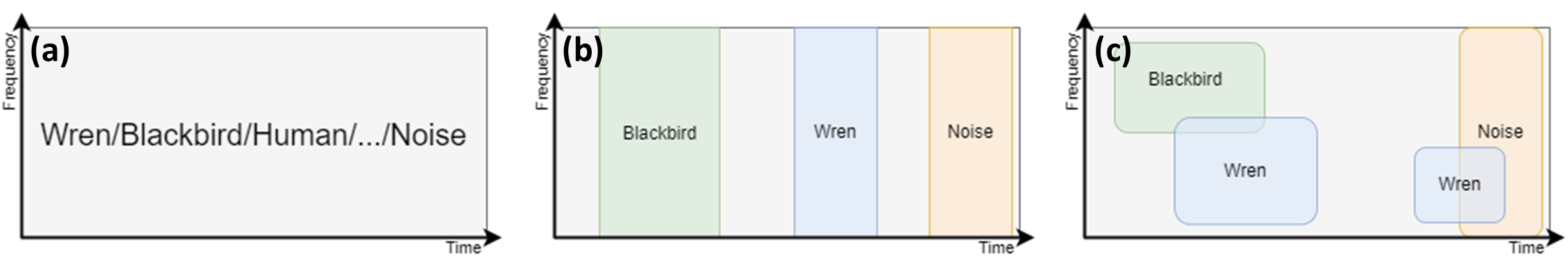

Supplement: Supplemental Information 5 [file peerj-11-15913-s005.png]

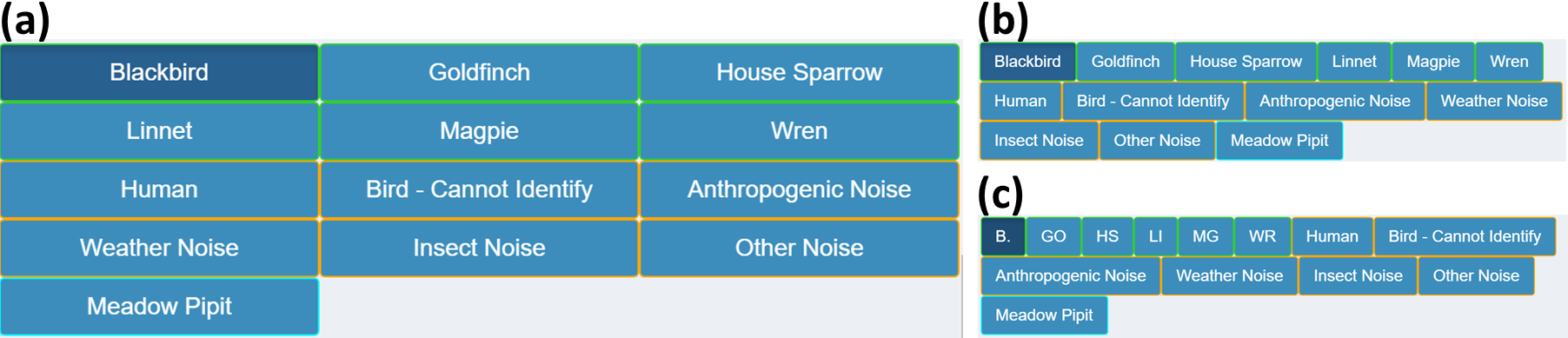

Supplement: Supplemental Information 6 [file peerj-11-15913-s006.png]

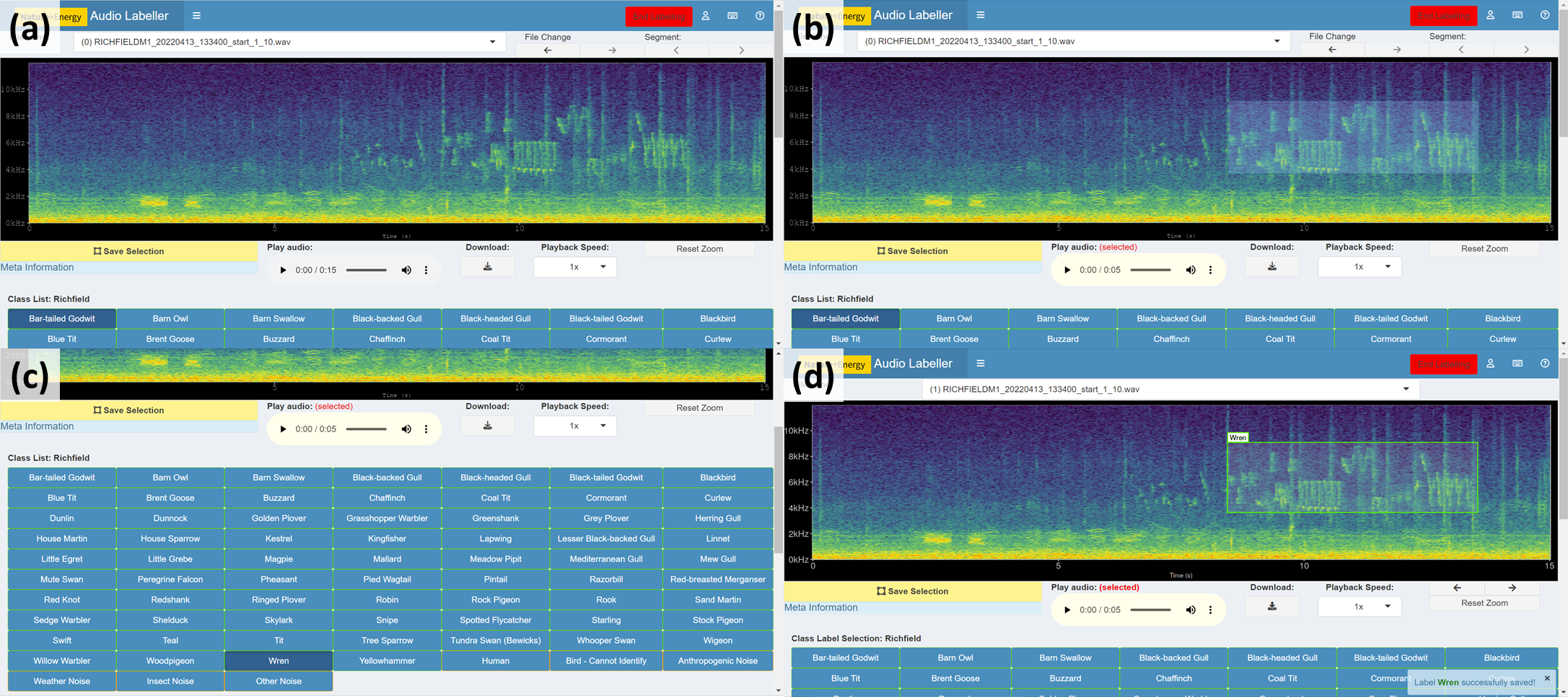

Supplement: Supplemental Information 7 [file peerj-11-15913-s007.png]

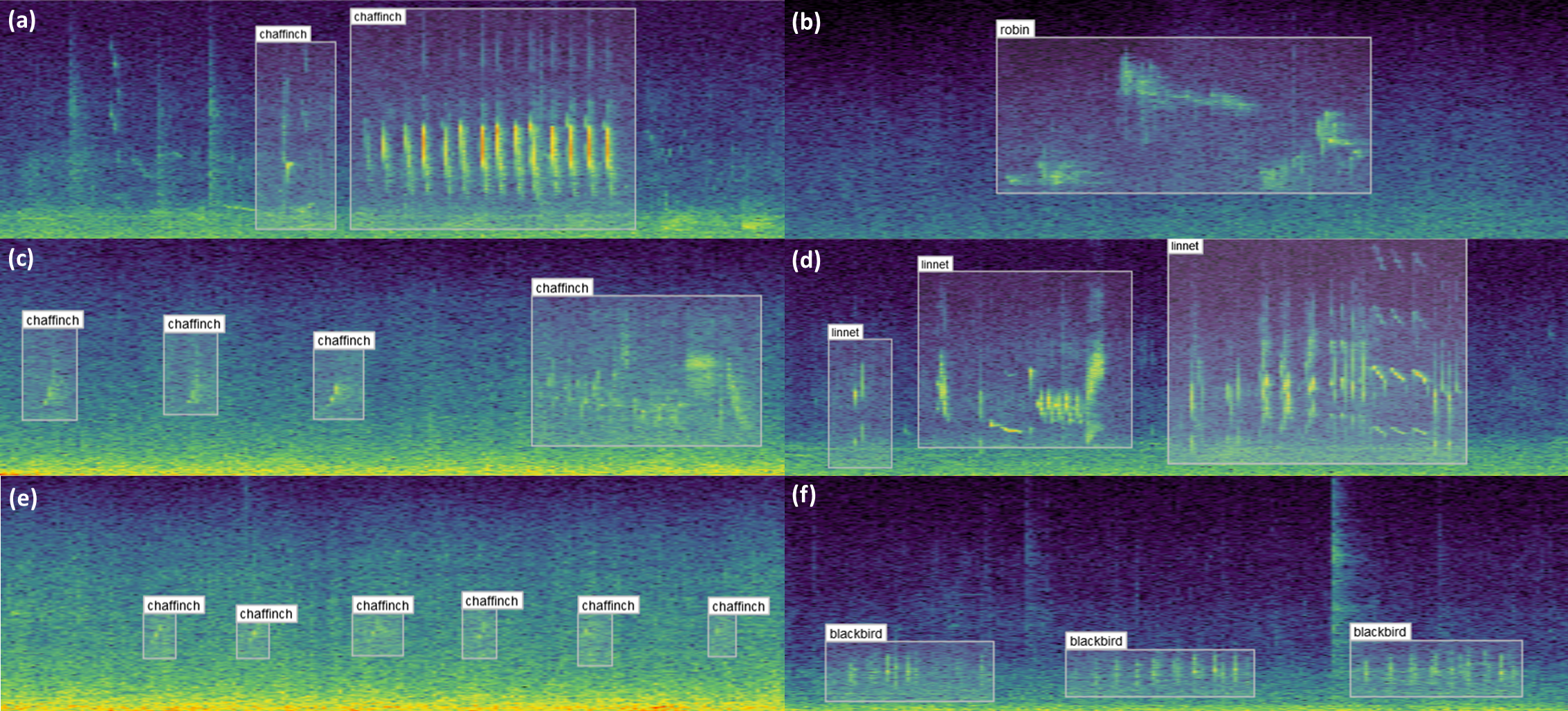

Supplement: Supplemental Information 8 [file peerj-11-15913-s008.png]

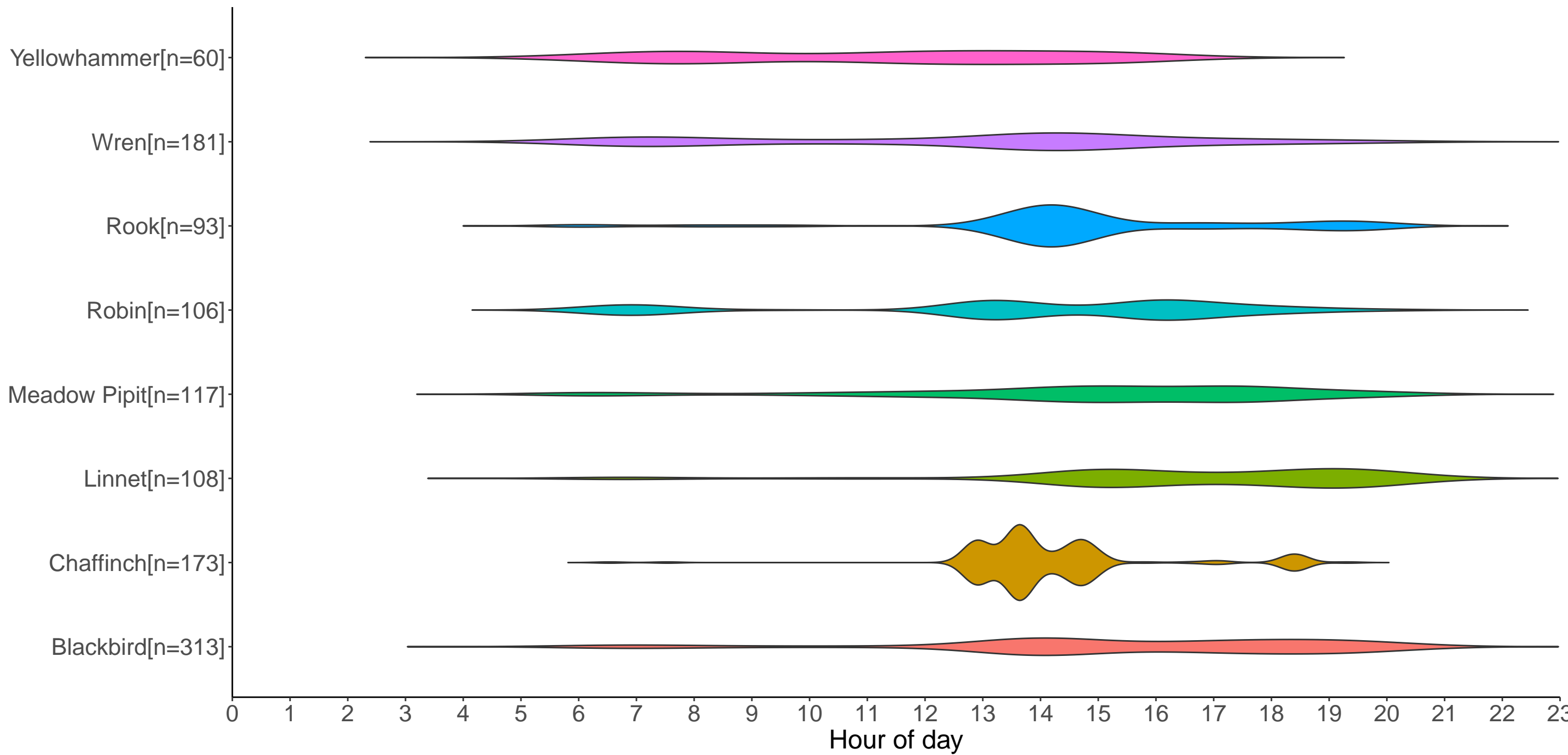

Supplement: Supplemental Information 9 [file peerj-11-15913-s009.pdf]
